# Dilated Cardiomyopathy: A Novel *BAG3* Mutation Associated with Aggressive Disease Progression and Ventricular Arrhythmias

**DOI:** 10.3390/jcdd12040121

**Published:** 2025-03-28

**Authors:** Paolo Pastori, Cristina Balla, Marta Rasia, Emilia Lo Jacono, Clelia Guerra, Roberta Schininà, Francesca Gualandi, Matteo Bertini, Giovanni Tortorella

**Affiliations:** 1 Cardiology Unit, Medical and Diagnostics Department, Fidenza Hospital, Azienda USL of Parma, 43036 Fidenza, Italy; ppastori@ausl.pr.it (P.P.); mrasia@ausl.pr.it (M.R.); elojacono@ausl.pr.it (E.L.J.); cguerra@ausl.pr.it (C.G.); gtortorella@ausl.pr.it (G.T.); 2 Cardiology Unit, Department of Translational Medicine, Sant’Anna University Hospital, University of Ferrara, Via Aldo Moro 8, 44124 Ferrara, Italy; bllcst@unife.it; 3 Medical Genetics Service, Department of Mother and Child, Sant’Anna University Hospital, Via Aldo Moro 8, 44124 Ferrara, Italy; roberta.schinina@unife.it (R.S.); gdf@unife.it (F.G.)

**Keywords:** dilated cardiomyopathy, *BAG3* gene mutation, ventricular arrhythmias, case report

## Abstract

We present the case of a 46-year-old man with a history of complex ventricular arrhythmias preceding the development of asymptomatic mild left ventricular dysfunction, who presented with acute-onset heart failure and was ultimately diagnosed with dilated cardiomyopathy. Genetic testing identified a novel, likely pathogenic mutation in exon 4 of the *BAG3* gene (NM_004281, c.1128del, (p.(Ser377AlafsTer47)), not previously reported in the literature. Given the presence of multiple clinical features indicative of a poor prognosis, he underwent prophylactic placement of a subcutaneous implantable cardioverter-defibrillator. The clinical presentation of this novel *BAG3* mutation suggests that it may be associated with a significant arrhythmic phenotype. This case underscores the importance of close follow-up and genetic testing in patients presenting with mild left ventricular dysfunction and ventricular arrhythmias.

## 1. Introduction

Dilated cardiomyopathy (DCM) is a myocardial disorder characterized by left or biventricular dilation and systolic dysfunction, with an estimated prevalence of approximately 1 in 250 individuals [1]. The degree of left ventricular (LV) systolic dysfunction in DCM varies and often progresses over time. Patients with DCM may remain asymptomatic for many years; however, when symptoms appear, the most common clinical presentations include heart failure (HF), arrhythmia, and thromboembolic events [2]. In approximately 50% of cases, a clear non-genetic cause can be identified, such as hypertension, coronary artery disease, valvular disease, or toxic exposures (e.g., alcohol or chemotherapeutic agents). However, in the remaining 50% of cases, genetic variants play a crucial role in disease pathogenesis. Although hundreds of genes have been associated with an increased risk of developing DCM, only twelve have demonstrated evidence of disease association [3,4]. In recent years, *BAG3* gene mutations have emerged as a significant genetic cause of DCM, classified among the definitive monogenic contributors to idiopathic DCM [5,6,7,8]. *BAG3* mutations are a relatively common cause of DCM, with a reported prevalence of 2.3% to 3.6% in affected cohorts [9]. The *BAG3* gene encodes *Bcl-2*-associated athanogene 3, an anti-apoptotic protein essential for excitation–contraction coupling and maintaining cellular homeostasis. DCM, associated with mutations in *BAG3*, is typically characterized by a high risk of progressive HF but is generally considered to carry a low arrhythmic risk [9]. Here, we describe a *BAG3* variant characterized by early-onset complex ventricular arrhythmia (VA) that preceded the development of overt DCM, suggesting a predominant arrhythmic phenotype.

## 2. Detailed Case Description

**Figure 1:**
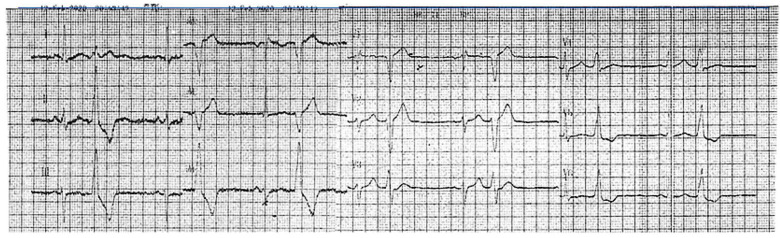
An ECG recorded in 2020 demonstrated sinus rhythm, incomplete right bundle branch block, and bigeminal PVCs with a left bundle branch block morphology and inferior axis.

A 46-year-old hypertensive male with a family history suggestive of cardiomyopathy (the mother died at 41 while awaiting a heart transplant) first presented in 2017 with frequent asymptomatic premature ventricular contractions (PVCs). A 24 h ECG Holter monitoring recorded 21,865 PVCs (25.3% burden), predominantly monomorphic with left bundle branch block morphology and an inferior axis (Figure 1).

**Figure 2:**
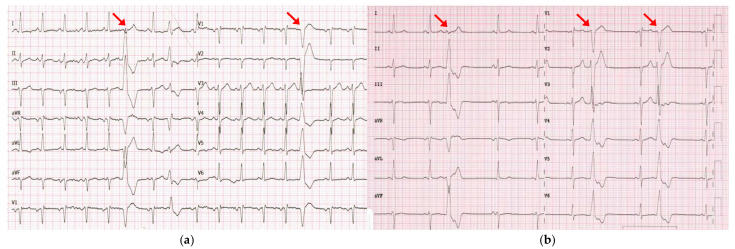
(**a**) ECG recorded in April 2023 during hospitalization for acute-onset HF showed sinus tachycardia and PVCs, mostly exhibiting the previously documented morphology (left bundle branch block with an inferior axis; red arrows). (**b**) ECG recorded in July 2023 documented the persistence of PVCs with the same morphology despite three months of optimal medical therapy.

Echocardiography at the time showed a structurally normal heart with normal left ventricular (LV) function and an ejection fraction (EF) of 60%. Even though flecainide effectively reduced the PVC burden to 1.8% (1526 PVCs), by 2021, the patient progressed to hypokinetic cardiomyopathy with a reduced EF of 45%. Consequently, flecainide was discontinued, and bisoprolol was initiated. The patient remained asymptomatic and declined recommended cardiac magnetic resonance imaging (MRI). In April 2023, he was admitted to our department with acute-onset HF. A physical examination revealed peripheral edema, crackles in both lungs and a heart rate of 102 beats per minute. Laboratory tests showed an elevated brain natriuretic peptide (BNP) level of 515 pg/mL. The electrocardiogram (ECG) showed sinus tachycardia, left anterior hemiblock, incomplete right bundle branch block (QRS 100 ms), and PVCs with different morphologies, most exhibiting the previously documented morphology (left bundle branch block with inferior axis) (Figure 2).

**Figure 3:**
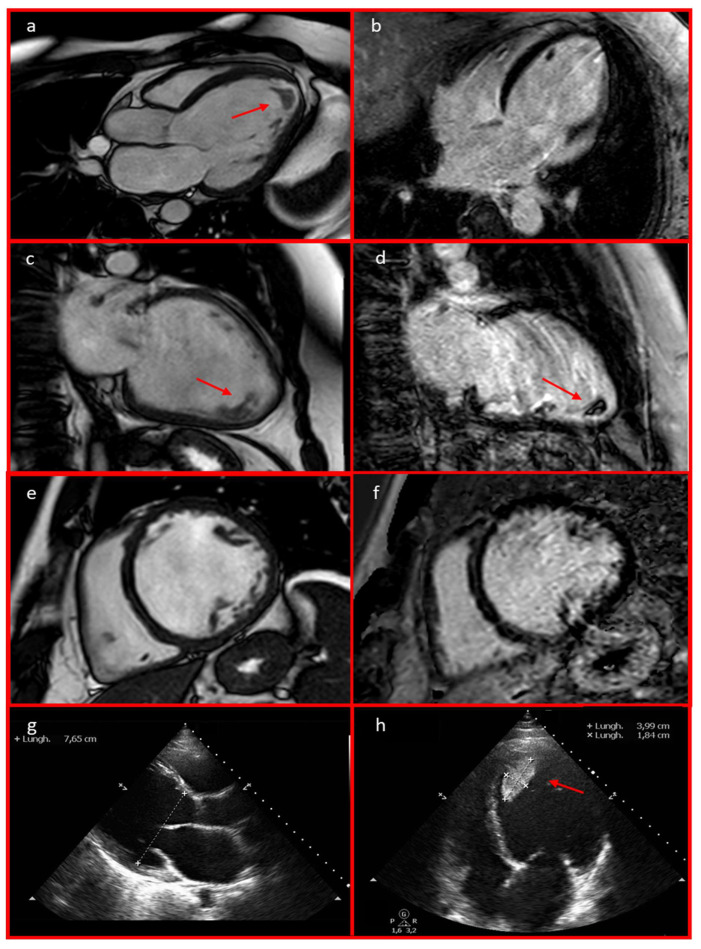
Cardiac MRI 3-chamber (**a**), 2-chamber (**c**), and short-axis (**e**) views revealed left ventricular dilation with severe biventricular dysfunction. These findings were also observed on the echocardiography in parasternal long-axis (**g**) and 4-chamber (**h**) views. An apical thrombus with a mobile component measuring 39.9 × 18.4 mm was identified (**a**,**c**,**d**,**h**; red arrows). Cardiac MRI late gadolinium enhancement (LGE) long-axis 4-chamber (**b**), 2-chamber (**d**), and short-axis (**f**) views excluded the presence of LGE.

**Figure 4:**
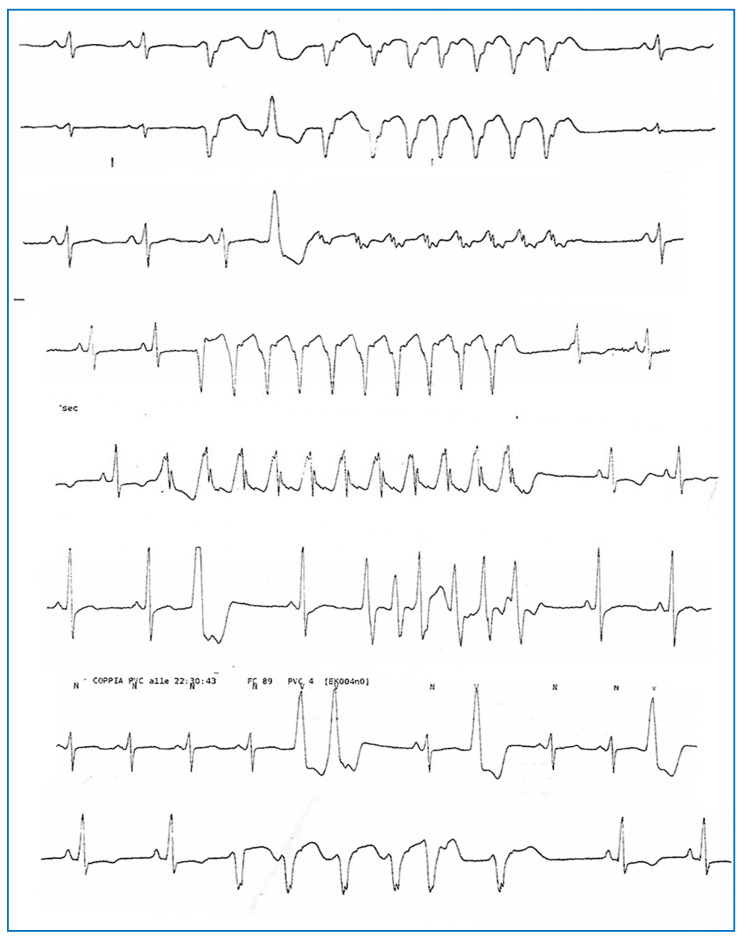
Complex ventricular arrhythmias (polymorphic NSVT, PVCs, and couplets) were recorded via telemetry monitoring during hospitalization in April 2023.

**Figure 5:**
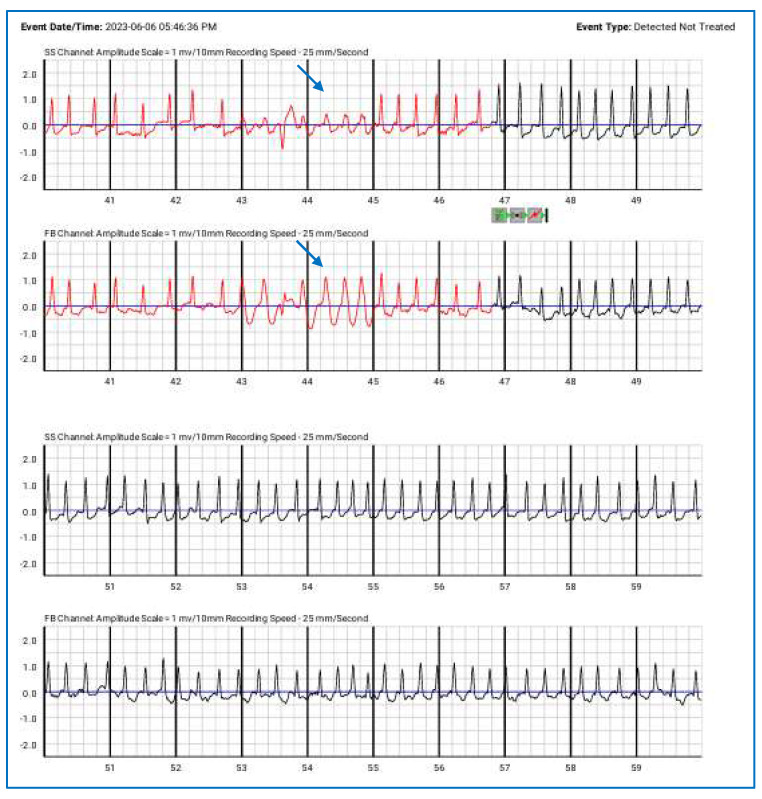
Episodes of paroxysmal atrial fibrillation correctly detected by the wearable cardioverter-defibrillator (detected but not treated), along with NSVT (blue arrows). The top lines represent the side-to-side (SS) channels, while the lower lines correspond to the front-to-back (FB) channels.

Echocardiography revealed severe left ventricular (LV) dilation with diffuse hypokinesia (LVEF 21%, left ventricular end-diastolic diameter [LVEED] 76 mm), moderate-to-severe functional mitral regurgitation, left atrial enlargement, and an apical thrombus with a mobile component (39.9 × 18.4 mm). These findings were confirmed by a cardiac MRI, which showed a dilated hypokinetic cardiomyopathy with severe biventricular dysfunction (LVEF 31%, left ventricular end-diastolic volume 164 mL/mq, right ventricular EF 40%, right ventricular end-diastolic volume 106 mL/mq). Notably, late gadolinium enhancement sequences were negative (Figure 3). A coronary CT angiography ruled out significant stenosis. Telemetry monitoring revealed polymorphic PVCs, along with couplets, triplets, and frequent episodes of non-sustained ventricular tachycardia (NSVT) (Figure 4). Guideline-directed medical therapy for the management of DCM was initiated, including valsartan, bisoprolol, furosemide, and canrenone. Anticoagulation therapy—enoxaparin bridged to warfarin—was administered for the apical thrombus. After achieving stable blood pressure levels, sacubitril/valsartan was introduced and titrated to the maximum tolerated dose (24/26 mg twice daily) over the following weeks. The patient was discharged with a wearable cardioverter-defibrillator (WCD) pending potential functional recovery, and the results of targeted genetic analysis for *LMNA* gene mutations guided the choice between subcutaneous or transvenous implantable cardioverter-defibrillator (ICD) implantation. During follow-up, remote monitoring via the WCD detected an episode of paroxysmal atrial fibrillation (Figure 5), along with PVCs and NSVT. In July 2023, after complete resolution of the apical thrombus and negative results from targeted genetic testing for *LMNA* mutations, the patient underwent S-ICD implantation. This decision was based on the patient’s young age, the presence of poor prognostic factors—such as male sex, reduced LVEF, and enlarged LVEDD [9]—and the persistence of severe ventricular dysfunction despite three months of optimal medical therapy.

**Figure 6:**
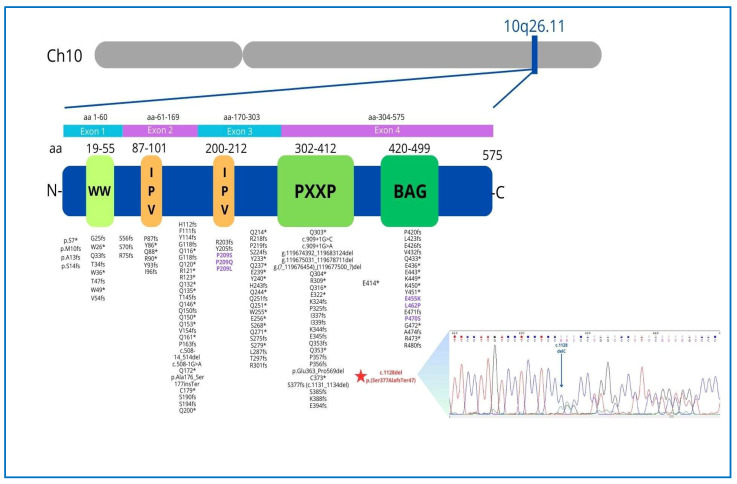
The *BAG3* gene, located on the long arm of chromosome 10 in the q26.11 region, encodes a 575 amino acid protein consisting of four functional domains with specific functions: the WW domain (amino acids 19–55) promoting integrin-mediated cell adhesion, 2 IPV motifs (amino acids 87–101, 200–213) involved in an interaction with Heat shock proteins (Hsp), the PXXP region (amino acids 302–412), mediating protein–protein interactions and the BAG domain (amino acids 420–499), which mediates the binding of *BAG3* with Hsp70 and binds Bcl-2 to inhibit apoptosis. The P/LP *BAG3* point variants archived in the Clinvar database (accession 19 January 2025) are reported (rare missense variants are indicated in purple). The asterisk (*) indicates that the mutation results in a stop codon, according to HGVS nomenclature, which is a universal standard. Chromatogram of the heterozygous *BAG3* c.1128del mutation (blue arrow) causing frameshifting.

**Figure 7:**
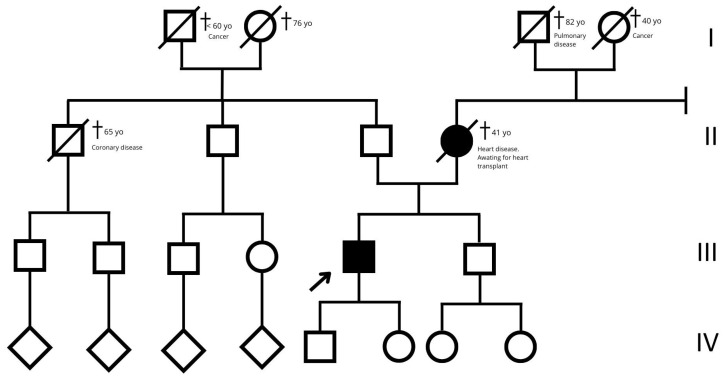
Family tree. The empty squares and circles indicate males and females, respectively. The corresponding filled-in symbols indicate the affected subjects in the family. The arrow indicates the proband. The crossed-out symbols indicate deceased subjects. Roman numerals symbolize generation. Related subjects are connected by horizontal or vertical lines, depending on the type of relationship.

Genetic testing using a custom next-generation sequencing cardiomyopathy gene panel (Table S1) identified a heterozygous frameshift mutation in exon 4 of the *BAG3* gene [NM_004281: c.1128del: p.(Ser377AlafsTer47)]. This novel mutation, absent from population databases, was classified as likely pathogenic (ACMG Class 4) [10] (Figure 6). The patient opted to postpone genetic testing for his children (15-year-old daughter and 13-year-old son) until they reached adulthood (Figure 7). Notably, their echocardiograms and ECG findings were unremarkable.

## 3. Discussion

The *BAG3* gene is located on chromosome 10q26.11, comprises four coding exons, and encodes *Bcl-2*-associated athanogene 3, a 575 amino acid protein that consists of four known functional domains, playing a fundamental role in excitation–contraction coupling and in maintaining cardiac cellular homeostasis.

*BAG3* is constitutively expressed in the heart, but it is also found in the skeletal muscle, brain, peripheral nervous system, and various forms of cancer [11]. It performs several cellular functions: inhibiting apoptosis via *Bcl-2* interaction, acting as a co-chaperone with heat shock proteins to facilitate autophagy, and preserving the structural stability of the sarcomere and the integrity of the Z-disk through the regulation of filamin clearance and by binding to the actin capping protein beta 1(CapZβ1) [8]. Mutations disrupt these functions, contributing to DCM pathogenesis. *BAG3* may, in fact, represent a crucial factor in cardiovascular pathophysiology, as decreased levels of BAG3 have been observed in end-stage non-familial failing myocardium, contributing to the pathogenesis of both familial and non-familial HF [12]. Cardiac histological data from the explanted heart tissue samples of patients with *BAG3* mutations who underwent heart transplantation displayed myofibril disarray and relocation of the BAG3 protein in the sarcomeric Z-disk, suggesting that DCM caused by the *BAG3* mutations is associated with a high risk of progressive heart failure [9].

**Figure 8:**
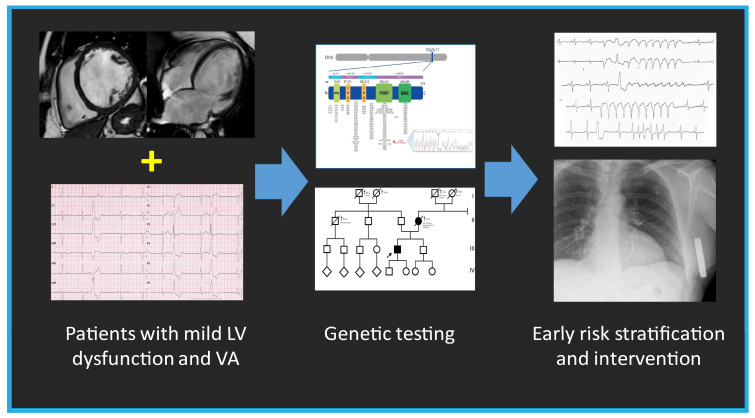
The summary figure (graphical abstract) emphasizes the importance of genetic testing in patients with mild left ventricular (LV) dysfunction and ventricular arrhythmia (VA), enabling early intervention and risk stratification.

The BAG3 protein plays a critical role in maintaining the stability of the cardiac sarcomere by interacting with the actin-capping protein CapZ and stabilizing the myofibrillar structure in response to mechanical stress. Consequently, alterations in BAG3 localization within the Z-disk may directly impair its function by disrupting its interaction with CapZ [11]. In contrast to neonatal myocytes, where BAG3 is found in the cytoplasm and involved in protein quality control and apoptosis, in adult LV myocytes, BAG3 is localized at the sarcolemma and T-tubules, modulating not only myocyte contraction but also action potential duration through specific interactions with the β1-adrenergic receptor and L-type Ca2+ channels. This provides important insights into the role of *BAG3* in cardiomyopathies and the increased risk of arrhythmias in HF [13]. The *BAG3* c.1128del novel mutation identified in our case consists of the deletion of a cytosine residue and is predicted to cause a translational frameshift with the introduction of 47 missense residues and the recognition of a premature stop codon within the BAG protein domain (Figure 6). A mutation with a similar protein consequence, consisting of a deletion of four nucleotides (c.1131_1134del (p.Ser377fs), is reported in the Clinvar Database (VCV002501612.3) without any clinical details. The c.1128del mutation occurs at the 3′ terminus of the *BAG3* gene and is not expected to trigger nonsense-mediated mRNA decay, thus probably producing a shorter protein. Since the first autosomal dominant *BAG3* mutation was reported in 2011 [5], numerous variants have been linked to DCM. Truncating mutations (nonsense and frameshifting) are largely predominant, with rare missense changes (Figure 6). These mutations carry a significant risk of progression to end-stage HF, with a 5.1% annual incidence of adverse cardiac events in overt DCM cases. Adverse outcomes are more common in males with reduced LVEF and LV dilation [9], as observed in our proband. While the annual incidence of VA in *BAG3* mutation carriers with a DCM phenotype is reported to be only 1.5% [9]—suggesting a lower arrhythmic risk than other genetic causes of DCM such as *LMNA* or *FLNC* mutations—this novel *BAG3* variant presented with complex VA, preceding the overt DCM phenotype by several years. This suggests a distinct arrhythmic phenotype, where frequent PVCs may serve as an early clinical marker before the onset of overt DCM, particularly in patients with mildly reduced LVEF (Figure 8). This study has some limitations. First, we did not have access to detailed information regarding the clinical course of the proband’s mother, including whether she experienced arrhythmia, as no clinical documentation was available. Second, definitive conclusions regarding the arrhythmogenic potential of *BAG3* cannot be drawn from a single case. A clear association between *BAG3* variants and arrhythmias remains to be established, particularly since previous studies have not identified a strong correlation.

## 4. Conclusions

In conclusion, the novel *BAG3* variant (c.1128del) exhibited a pronounced arrhythmic phenotype, with complex VA notably preceding the rapid progression to severe non-scarring DCM. These findings have significant clinical implications. First, they expand our understanding of DCM pathogenesis and highlight the phenotypic heterogeneity of *BAG3*-related cardiomyopathy. Second, they underscore the importance of genetic testing in patients with mild ventricular dysfunction and VA, enabling early intervention and risk stratification. Finally, they emphasize the need for close monitoring and individualized management strategies in *BAG3* mutation carriers, including the consideration of ICD placement in young patients with *BAG3*-related DCM to mitigate sudden cardiac death risk.

## Data Availability

The data underlying this article will be shared upon reasonable request to the corresponding author.

## Supplementary Materials

The following supporting information can be downloaded at https://www.mdpi.com/article/10.3390/jcdd12040121/s1. Table S1: Cardiomyopathy gene panel.

## Abbreviations

The following abbreviations are used in this manuscript:

DCM	dilated cardiomyopathy
*BAG3*	*BCL2*-associated athanogene 3
HF	heart failure
VA	ventricular arrhythmias
PVCs	premature ventricular contractions
LV	left ventricular
EF	ejection fraction
ECG	electrocardiogram
MRI	magnetic resonance imaging
LVEDD	left ventricular end-diastolic diameter
NSVT	non-sustained ventricular tachycardia
WCD	wearable cardioverter-defibrillator
*LMNA*	Lamin A/C protein-coding gene
S-ICD	subcutaneous implantable cardioverter-defibrillator
