# Supplementary material for: Dilated Cardiomyopathy: A Novel BAG3 Mutation Associated with Aggressive Disease Progression and Ventricular Arrhythmias
Source: J Cardiovasc Dev Dis. 2025 Mar 28;12(4):121. doi: 10.3390/jcdd12040121 (PMC12027914; doi:10.3390/jcdd12040121)
Supplement: Supplementary file 1 [file jcdd-12-00121-s001.zip › jcdd-3515002-supplementary.pdf]

Supplementary Table S1

Cardiomyopathy Gene Panel

ABCC9 (NM_005691)	GAA (NM_000152)	PLN (NM_002667)
ACTC1 (NM_005159)	GLA (NM_000169)	PRKAG2 (NM_016203)
ACTN2 (NM_001103)	JPH2 (NM_020433)	RAF1 (NM_002880)
ANKRD1 (NM_014391)	JUP (NM_002230)	RBM20 (NM_001134363)
BAG3 (NM_004281)	LAMP2 (NM_002294)	SCN5A (NM_198056)
CASQ2 (NM_001232)	LDB3 (NM_007078)	SGCD (NM_000337)
CAV3 (NM_033337)	LMNA (NM_170707)	TAZ (NM_000116)
CRYAB (NM_001885)	MYBPC3 (NM_000256)	TCAP (NM_003673)
CSRP3 (NM_003476)	MYH6 (NM_002471)	TNNC1 (NM_003280)
DES (NM_001927)	MYH7 (NM_000257)	TNNI3 (NM_000363)
DMD (NM_004006)	MYL2 (NM_000432)	TNNT2 (NM_001001430)
DSC2 (NM_024422)	MYL3 (NM_000258)	TPM1 (NM_001018005)
DSG2 (NM_001943)	MYLK2 (NM_033118)	TTN (NM_001267550 esoni 1-175,199-364)
DSP (NM_004415)	MYOZ2 (NM_016599)	TTR (NM_000371)
DTNA (NM_001390)	MYPN (NM_032578)	VCL (NM_014000).
FKTN (NM_001079802)	NEXN (NM_144573)	
FLNC (NM_001458)	PKP2 (LOVD NM_004572MANE Select NM_001005242)	
